# DeepHBV: a deep learning model to predict hepatitis B virus (HBV) integration sites

**DOI:** 10.1186/s12862-021-01869-8

**Published:** 2021-07-07

**Authors:** Canbiao Wu, Xiaofang Guo, Mengyuan Li, Jingxian Shen, Xiayu Fu, Qingyu Xie, Zeliang Hou, Manman Zhai, Xiaofan Qiu, Zifeng Cui, Hongxian Xie, Pengmin Qin, Xuchu Weng, Zheng Hu, Jiuxing Liang

**Affiliations:** 1 Institute for Brain Research and Rehabilitation, South China Normal University, Guangzhou, 510631 Guangdong China; 2 Department of Medical Oncology of the Eastern Hospital, the First Affiliated Hospital, Sun Yat-Sen University, Guangdong 510700 Guangzhou, China; 3 Department of Gynecological Oncology, the First Affiliated Hospital, Sun Yat-Sen University, Guangdong 510080 Guangzhou, China; 4 Department of Thoracic Surgery, the First Affiliated Hospital, Sun Yat-Sen University, Guangdong 510080 Guangzhou, China; 5 School of Psychology, South China Normal University, Guangzhou, 510080 Guangdong China; 6 Generulor Company Bio-X Lab, Guangzhou, 510006 Guangdong China; 7 Key Laboratory of Brain, Cognition and Education Sciences (South China Normal University), Ministry of Education, Guangzhou, 510080 Guangdong China; 8 Department of Obstetrics and Gynecology, Tongji Hospital, Tongji Medical College, Huazhong University of Science and Technology, Wuhan, 430030 Hubei China; 9 School of Computer Science, South China Normal University, Guangzhou, 510631 China

**Keywords:** Deep learning, HBV integration sites, Genomic features, Bioinformatics

## Abstract

**Background:**

The hepatitis B virus (HBV) is one of the main causes of viral hepatitis and liver cancer. HBV integration is one of the key steps in the virus-promoted malignant transformation.

**Results:**

An attention-based deep learning model, DeepHBV, was developed to predict HBV integration sites. By learning local genomic features automatically, DeepHBV was trained and tested using HBV integration site data from the dsVIS database. Initially, DeepHBV showed an AUROC of 0.6363 and an AUPR of 0.5471 for the dataset. The integration of genomic features of repeat peaks and TCGA Pan-Cancer peaks significantly improved model performance, with AUROCs of 0.8378 and 0.9430 and AUPRs of 0.7535 and 0.9310, respectively. The transcription factor binding sites (TFBS) were significantly enriched near the genomic positions that were considered. The binding sites of the AR-halfsite, Arnt, Atf1, bHLHE40, bHLHE41, BMAL1, CLOCK, c-Myc, COUP-TFII, E2A, EBF1, Erra, and Foxo3 were highlighted by DeepHBV in both the dsVIS and VISDB datasets, revealing a novel integration preference for HBV.

**Conclusions:**

DeepHBV is a useful tool for predicting HBV integration sites, revealing novel insights into HBV integration-related carcinogenesis.

**Supplementary Information:**

The online version contains supplementary material available at 10.1186/s12862-021-01869-8.

## Background

HBV is the main cause of viral hepatitis and liver cancer (HCC) [1]. HBV can integrate into the host genome via an RNA intermediate due to its small size [1]. Cases of viral DNA integrated into the human genome were detected in 85–90% of HBV-related HCCs [2]. HBV attaches and enters hepatocytes, then transports its nucleocapsid, which contains a relaxed circular DNA (rcDNA), to the host nucleus. In the host nucleus, rcDNA is converted into covalently closed circular DNA (cccDNA), which produces messenger RNA (mRNA) and pre-genomic RNA (pgRNA) by transcription. Then, pgRNA produces new rcDNA and double-stranded linear DNA (dslDNA) via reverse transcription in the host nucleus, which tend to integrate into the host cell genome [3].

A previous study showed HBV integration breakpoints distributed randomly across the whole genome with a handful of hotspots [7]. Further analysis revealed an association between HBV integration and genomic instability during these insertional events [8]. Moreover, significant enrichment of HBV integration was found near the following genomic features: repetitive regions, fragile sites, CpG islands, and telomeres in tumors compared to non-tumor tissues [3]. For instance, HBV integration was reported to recur in the telomerase reverse transcriptase (*TERT*) and myeloid/lymphoid or mixed-lineage leukemia 4 (*MLL4*, also known as *KMT2B*) genes. The insertional events were also accompanied by altered expression of the integrated gene [3, 7, 9], indicating important biological impacts on the local genome. However, the pattern and mechanism of HBV integration remain to be explored. Many HBV integration sites are distributed throughout the human genome and seem completely random [8, 10, 11]. Whether the features and patterns of these “random” viral integration events could be learned and extracted remains an open question, and once solved, will greatly improve the understanding of HBV integration-related carcinogenesis.

Deep learning has shown a promising ability to discover intricate structures in multidimensional data and automatically extract features from these data [12]. Under the conditions of big data and a well-designed network structure, deep learning models can better predict performance in many cases. Moreover, deep learning has performed excellently in computational biology research such as medical image identification [13] and protein sequence motif discovery [14]. The convolutional neural network (CNN) is the most important part of deep learning, enabling a computer to learn and program itself from training data [15]. Although deep learning performs well in various fields, the detailed theory of how it makes decisions is difficult to explain due to its black box effect. Therefore, an approach called the attention mechanism, which can highlight the outstanding parts and connect the encoder and the decoder was invented to open the “black box” [16, 17].

This study developed DeepHBV, an attention-based model for predicting HBV integration sites using deep learning. The attention mechanism highlights the regions concentrated upon by DeepHBV and helps determine the investigated patterns. DeepHBV can predict HBV integration sites accurately and specifically, and the attention mechanism highlights positions with potentially important biological meanings. Our work identified novel transcription factor-binding sites (TFBSs) near HBV integration hotspots, revealing new insights into HBV-induced cancer.

## Results

### DeepHBV effectively predicts HBV integration sites by adding genomic features

**Fig. 1:**
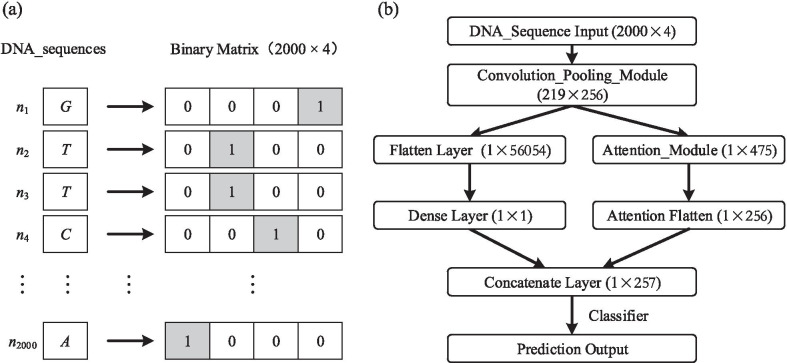
The deep learning framework applied in DeepHBV. (**a**) Scheme of encoding a 2 kb DNA sequence into a binary matrix using one-hot code; (**b**) A brief flowchart of DeepHBV structure, the matrix shape was included in brackets, and a detailed flowchart was in Supplementary Figure 1

**Fig. 2:**
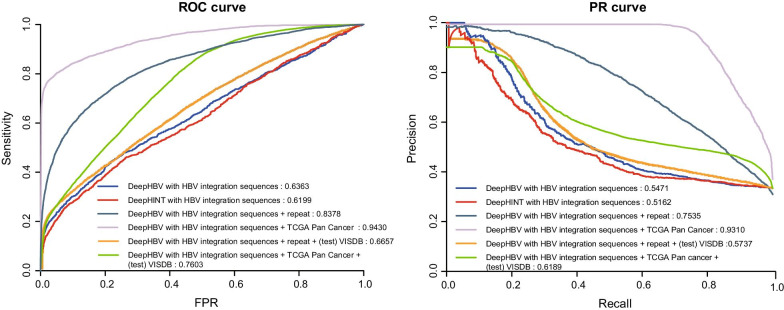
Evaluation of DeepHBV model prediction performance on the test dataset: (**a**) receiver-operating characteristic (ROC) curves and (**b**) precision recall (PR) curves, respectively

The DeepHBV model structure and the scheme of encoding a 2000 bp sample into a binary matrix are shown in Fig. 1. The DeepHBV model was tested using the HBV integration sites database (http://dsvis.wuhansoftware.com). HBV integration sequences were prepared according to HBV integration sites as positive samples, following the steps in the method. The negative sample abstracting also followed the method, and the negative samples should be twice the number of positive samples to maintain data balance and improve the confidence level. The positive samples were divided into 2902 and 1264 positive training datasets and testing datasets, respectively. We extracted 5804 and 2528 negative samples as the negative training dataset and testing dataset, respectively. Tests were performed on the DeepHBV model using samples of DNA sequences near the HBV integration sites. DeepHINT, an existing deep learning model for predicting HIV integration sites according to the surroundings [18], was also evaluated using HBV integration sequences for training and testing. The preparation of input data for DeepHINT also applied 2000 bp sequences near HIV integration sites [18]. We tested DNA sequence samples with lengths of 500 bp, 1000 bp, and 2000 bp and 4000 bp. As shown in Additional file 4: Table S5, 2000 bp had the largest accuracy (0.7368), sensitivity (0.7695), specificity (0.7321), AUROC (0.6901) and the most of the performance results among all tested lengths. In this case, 2000 bp sequences were used in our study. The ReLU activation function and the almost identical encoding function make it possible to use HBV integration sequences to perform tests on DeepHINT. Both models were trained using the same HBV integration training dataset, and the same testing dataset was used for the evaluation. The results showed that DeepHBV with HBV integration sequences had an AUROC of 0.6363 and an AUPR of 0.5471, while DeepHINT with HBV integration sequences had an AUROC of 0.6199 an AUPR of 0.5152 (Fig. 2). Except for AUROC and AUPR, the tenfold cross-validation and confusion matrix, which included true positives, true negatives, false positives, and false negatives, followed by accuracy, specificity, sensitivity, Mathews’ correlation coefficient, and F−1 score were applied to evaluate the predictive model, and the results are shown in Table 1 and Additional file 4: Table S8.

**Table 1:** The testing results of DNA sequence samples of 2000 bp length

Fold No	Loss	Accuracy	Sensitivity	Specificity	AUROC	AUPR	F1-score	MCC
1	1.1799	0.7518	0.7919	0.7452	0.7204	0.6365	0.4752	0.3983
2	1.2281	0.7473	0.7892	0.7407	0.7084	0.6293	0.4585	0.3855
3	1.1282	0.7451	0.7799	0.7396	0.7014	0.6198	0.4534	0.3780
4	1.2226	0.7146	0.7344	0.7125	0.6588	0.5595	0.3326	0.2809
5	1.0598	0.7178	0.7270	0.7167	0.6627	0.5574	0.3553	0.2915
6	1.1714	0.7279	0.7812	0.7217	0.6689	0.5720	0.3749	0.3269
7	1.1656	0.7425	0.7538	0.7406	0.7027	0.6195	0.4582	0.3695
8	1.3303	0.7486	0.8125	0.7393	0.7104	0.6309	0.4508	0.3905
9	0.7810	0.7241	0.7294	0.7234	0.6442	0.5477	0.3876	0.3124
10	1.0883	0.7483	0.7957	0.7409	0.7232	0.6389	0.4580	0.3881
Mean	1.1355	0.7368	0.7695	0.7321	0.6901	0.6012	0.4204	0.3521
SD	0.1385	0.0134	0.0293	0.0114	0.0271	0.0352	0.0494	0.0424

*AUROC* area under receiver operating characteristic curve; *AUPR* area under precision-recall curve; *MCC* Mathews’ correlation coefficient

Several previous studies have shown that HBV integration prefers surrounding genomic features such as repeats, histone markers, CpG islands, among other features [3, 8]. Thus, we added these genomic features into DeepHBV by mixing some genomic feature samples with HBV integration sequences as new datasets and then trained and tested the updated DeepHBV models. We downloaded the following genomic features from different datasets [19–21] into four subgroups: (1) DNase Clusters, Fragile site, RepeatMasker; (2) CpG islands, GeneHancer; (3) Cons 20 Mammals, TCGA Pan-Cancer; (4) H3K4Me3 ChIP-seq, H3K27ac ChIP-seq (Additional file 2: Fig. S2a, b). After obtaining genomic feature data positions (sources are mentioned in Additional file 4: Table S2), we extended the positions to 2000 bp and extracted related sequences on the hg38 reference genome. These sequences were defined as positive genomic feature samples. We then mixed HBV integration sequences, positive genome feature samples, randomly picked negative genomic feature samples (see Method), 0, and trained the DeepHBV model. Once a subgroup performed well, we re-tested each genomic feature in that subgroup to determine which specific genomic features significantly affected the model performance (Additional file 2: Fig. S2) (AUROC and AUPR values were recorded in Additional file 4: Table S3). From the ROC and PR curves, we found that DeepHBV with HBV integration sites and the genomic features repeat (AUROC: 0.8378 and AUPR: 0.7535) and TCGA Pan Cancer (AUROC: 0.9430 and AUPR: 0.9310) can significantly improve the HBV integration site prediction performance against DeepHBV with HBV integration sequences (Fig. 2). We also performed the same test on DeepHINT but did not find a subgroup that could substantially improve the model performance (these results are recorded in Additional file 4: Table S3). Thus, DeepHBV with HBV integration sequences plus repeat or TCGA Pan Cancer can significantly improve model performance.

### Validation of DeepHBV using the VISDB independent dataset

DeepHBV must be applied to general datasets, so we tested the pre-trained DeepHBV models (DeepHBV with HBV integration sequences + repeat peaks and DeepHBV with HBV integration sequences + TCGA Pan-Cancer peaks) on the HBV integration sites dataset in another virus integration sites (VIS) database, VISDB [22]. We found that in the model trained with HBV integration sequences + repeat sequences, the AUROC and AUPR were 0.6657 and 0.5737, respectively, while the model trained with HBV integrated sequences + TCGA Pan Cancer showed an AUROC of 0.7603 and an AUPR of 0.6189.

The DeepHBV model with HBV integration sequences + TCGA Pan Cancer performed better than the DeepHBV model with HBV integration sequences + repeat and was more robust for both testing datasets from dsVIS (AUROC: 0.9430 and AUPR: 0.9310) and the independent testing dataset from VISDB (AUROC: 0.7603 and AUPR: 0.6189). Thus, we decided to use this model for future HBV integration site predictions. With repeat or TCGA Pan-Cancer genome features, the probability of a 2000 bp input DNA sequence to be an HBV integration site in the human genome can be predicted accurately by DeepHBV.

### Study HBV integration site selection preference by important sequence elements

**Fig. 3:**
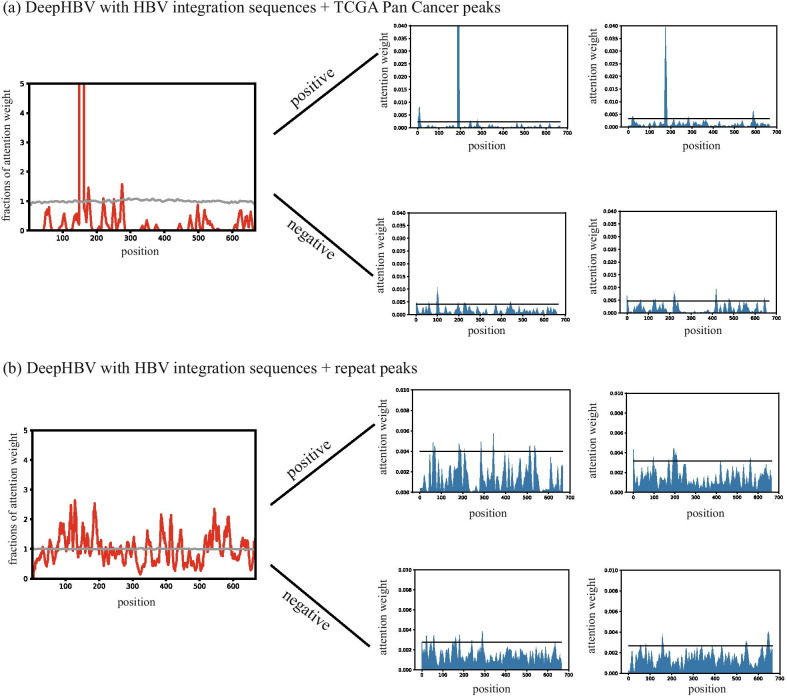
The attention weight distribution of analysed by DeepHBV with HBV integration sequences + genomic features. (**a**) DeepHBV with HBV integration sequences + TCGA Pan Cancer peaks; (**b**) DeepHBV with HBV integration sequences + repeat peaks. The left graph showed the fractions of attention weight, which were averaged among all samples and normalized to the average of all positions, each index represents a 3 bp region due to the multiple convolution and pooling operation. The graphs on the right are representative samples of attention weight distribution of positive samples and negative samples

DeepHBV can extract features with translation invariance by pooling operations, enabling DeepHBV to recognize certain patterns even when the features are slightly translated. The participation of the attention mechanism in the DeepHBV framework might partly open the deep learning black box by giving attention to each position. Each attention weight represented the computational importance level of that position in the DeepHBV judgment. The attention weights in the attention layer were extracted after two de-convolutions and one de-pooling operation, and the output shape was 667 × 1. Each value represents the attention weight of a 3 bp region. Positions with higher attention weight values might have a more important impact on the pattern recognition of DeepHBV, which means these positions might be the critical points for identifying HBV integration sites. We defined the fractions of attention values averaged among each site of all HBV integration sequences and normalized them to the mean of all positions. We then visualized the fractions of identified attention values where the figure showed peak-valley-peak patterns only in positive samples (Fig. 3). We were interested in the positions with higher attention weights, which were monitored in the CNN. In addition, we found that in the attention weight distribution of DeepHBV with HBV integration sites + TCGA Pan-Cancer, a cluster of attention weights that were much higher than other weights in the same sample often occurred in positive samples. However, in the model of DeepHBV with HBV integration sites + repeats, this pattern was not observed (Fig. 3).

**Fig. 4:**
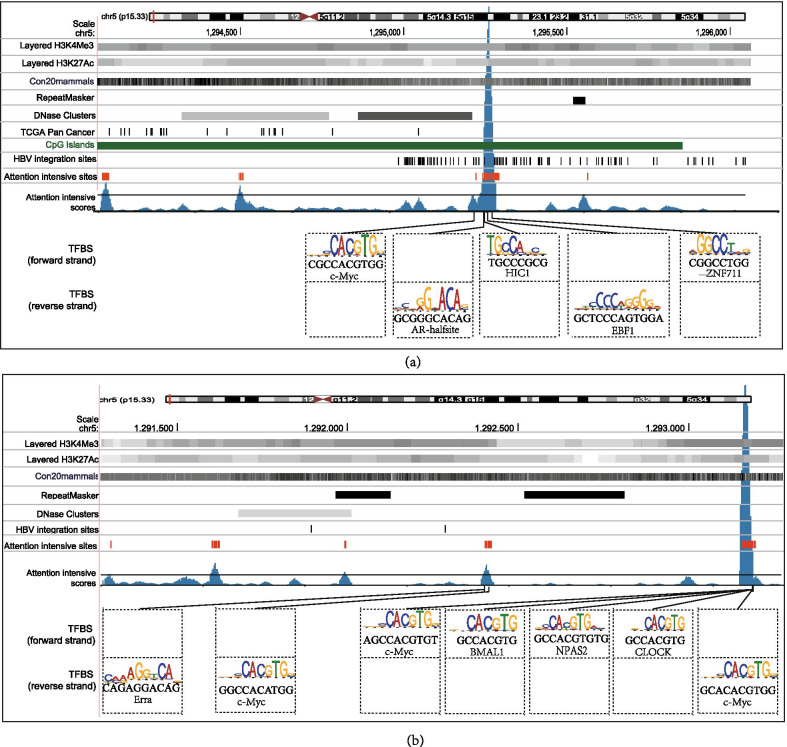
Attention intensive regions highlighted essential local genomic features on predicting HBV integration sites. Representative examples showed the positional relationship between the attention intensive sites and several genomic features using DeepHBV with HBV integration sequences + TCGA Pan Cancer model on (**a**) chr5:1,294,063-1,296,063 (hg38), (**b**) chr5: 1291277-1293277 (hg38)

To further discover the pattern behind these positions with higher attention weights, we defined the sites with the top 5% highest attention weights as attention-intensive sites and the regions of 10 bp near them as attention-intensive regions. We mapped these attention-intensive sites on the hg38 reference genome with genomic features (Fig. 4) but found that the positional relationship between attention-intensive sites and genomic features was not quite clear.

The convolution and pooling module learns the patterns with translation invariance in deep learning, based on the fact that the deep learning network tends to learn the domains that occur recurrently among samples within the same pooling matrix, even though the learned feature was not at the same position in different samples [23, 24]. Attention-intensive regions are more likely to be conserved because of translation invariance in the convolution and pooling modules. The accurate results indicated that the conserved regions would provide hints to the selection preference of HBV integration sites. We then designed and applied an attempt to enrich transcription factor-binding site (TFBS) motifs, conserved genomic elements in these attention-intensive regions. We used all HBV integration samples with prediction values higher than 0.95 from dsVIS and VISDB separately to enrich local TFBS motifs in attention-intensive regions using HOMER v 4.11.1 [25] with the vertebrate transcription factor databases provided by HOMER. We enriched several TFBS near attention-intensive sites, which are shown in Table 2. From the result of DeepHBV with HBV integration sequences + TCGA Pan-Cancer, the binding sites of AR-halfsite, Arnt, Atf1, bHLHE40, bHLHE41, BMAL1, CLOCK, c-Myc, COUP-TFII, E2A, EBF1, Erra, Foxo3, HEB, HIC1, HIF-1b, LRF, Meis1, MITF, MNT, Myoga, n-Myc, NPAS2, NPAS, Nr5a2, Ptf1a, Snail1, Tbx5, Tbx6, TCF7, TEAD1, TEAD3, TEAD4, TEAD, Tgif1, Tgif2, THRb, USF1, Usf2, Zac1, ZEB1, ZFX, ZNF692, ZNF711 can be enriched in both attention-intensive regions of dsVIS and VISDB sequences. We selected two representative samples to obtain a more intuitive display. Genomic features, HBV integration sites from dsVIS and VISDB, attention-intensive sites, and TFBS were aligned and shown in the hg38 reference genome (Fig. 4). Most attention-intensive sites can be mapped to enrich the TF motifs. The clusters of high attention weight from the output of DeepHBV with HBV integration sites plus TCGA Pan-Cancer in Fig. 4 show the binding site of a tumor suppressor gene *HIC1* or circadian clock-related elements BMAL1, CLOCK, c-Myc, and NAPS2. Together, the data provided novel insights into HBV integration site selection preference and revealed biological importance that warrants future experimental confirmation.

**Table 2:** Enriched TFBS from attention intensive regions of DeepHBV with HBV integration sites + TCGA Pan Cancer peaks

HOMER known results			HOMER de novo results		
Rank	Name	P-value	Rank	Best match/details	P-value
1	BMAL1	1E−323	1	TEAD3	1E−2283
2	NPAS	1.00E−259	2	EBF1	1E−1926
3	CLOCK	1.00E−165	3	TCF7	1E−958
4	c−Myc	1.00E−126	4	GRHL2	1E−504
5	ZFX	1.00E−108	5	Dux	1E−477
6	Tgif2	1.00E−75	6	Ptf1a	1E−465
7	MNT	1.00E−71	7	TEAD	1E−385
8	LRF	1.00E−62	8	Ahr::Arnt	1.00E−302
9	Tbx5	1.00E−62	9	Sox5	1.00E−245
10	ZNF711	1.00E−57	10	TEAD	1.00E−233
11	n-Myc	1.00E−54	11	Zic2	1.00E−204
12	ZNF416	1.00E−52	12	Nr2e3	1.00E−197
13	USF1	1.00E−47	13	SOX18	1.00E−182
14	bHLHE40	1.00E−45	14	ZBTB14	1.00E−174
15	Rbpj1	1.00E−36	15	USF2	1.00E−153
16	Zac1	1.00E−35	16	Isl1	1.00E−142
17	Tgif1	1.00E−32	17	ZNF264	1.00E−142
18	ZEB1	1.00E−30	18	Ascl2	1.00E−133
19	THRb	1.00E−29	19	ZNF460	1.00E−120
20	Ptf1a	1.00E−29	20	LRF	1.00E−117
21	bHLHE41	1.00E−29	21	ZNF416	1.00E−117
22	TEAD1	1.00E−27	22	PKNOX1	1.00E−103
23	Stat3	1.00E−24	23	Bcl6b	1.00E−91
24	Meis1	1.00E−21	24	Arnt	1.00E−90
25	c-Myc	1.00E−21	25	Osr2	1.00E−88
26	Usf2	1.00E−20	26	TFAP2A	1.00E−79
27	NPAS2	1.00E−17			
28	HIC1	1.00E−17			
29	TEAD	1.00E−17			
30	TEAD4	1.00E−16			
31	AR-halfsite	1.00E−16			
32	STAT6	1.00E−15			
33	TCF4	1.00E−13			
34	MITF	1.00E−13			
35	TEAD3	1.00E−13			
36	Atf1	1.00E−12			
37	HIF-1b	1.00E−11			
38	Foxo3	1.00E−10			
39	E2A	1.00E−09			
40	TEAD2	1.00E−09			
41	Mef2a	1.00E−08			
42	ZNF692	1.00E−07			
43	Nkx3.1	1.00E−07			
44	COUP-TFII	1.00E−07			
45	MyoG	1.00E−07			
46	Nkx2.5	1.00E−06			
47	Snail1	1.00E−05			
48	HEB	1.00E−05			
49	Tbx6	1.00E−05			
50	SCRT1	1.00E−04			
51	Nr5a2	1.00E−04			
52	Nanog	1.00E−03			
53	Oct11	1.00E−03			
54	Elk1	1.00E−03			
55	Erra	1.00E−03			
56	Gata6	1.00E−03			
57	BHLHA15	1.00E−03			
58	AMYB	1.00E−03			
59	Nr5a2	1.00E−03			
60	NFkB-p65-Rel	1.00E−02			
61	Zic	1.00E−02			
62	TRPS1	1.00E−02			
63	Hoxa9	1.00E−02			
64	HIF2a	1.00E−02			
65	Isl1	1.00E−02			
66	CEBP:AP1	1.00E−02			
67	EWS:FLI1-fusion	1.00E−02			
68	FOXK1	1.00E−02			
69	ETS	1.00E−02			

## Discussion

This study developed an explainable attention-based deep learning model, DeepHBV, to predict HBV integration sites. In comparing DeepHBV and DeepHINT for predicting HBV integration sites (Additional file 4: Table S3), DeepHBV outperformed DeepHINT after adding genomic features due to its more suitable model structure and parameters for recognizing the surroundings of HBV integration sites. We applied two convolution layers (first layer: 128 convolution kernels with a kernel size of 8; second layer: 256 convolution kernels with a kernel size of 6) and one pooling layer (with a pooling size of 3) in DeepHBV. In DeepHINT, the model only has one convolution layer (64 convolution kernels with a kernel size of 6) and one pooling layer (with a pool size of 3). Increasing the convolution layers enables the information from higher dimensions to be extracted, and the increase in convolution kernels enables more feature information to be extracted [26].

We trained the DeepHBV model using three strategies: (1) DNA sequences near HBV integration sites (HBV integration sequences), (2) HBV integration sequences + TCGA Pan-Cancer peaks, and (3) HBV integration sequences + repeat peaks. We found that the model with HBV integration sequences adding TCGA Pan Cancer or repeats can significantly improve model performance. The DeepHBV with HBV integration sequences adding TCGA Pan-Cancer peaks performed better with the VISDB independent test dataset. However, attention-intensive regions cannot be well-aligned to these genomic features. Thus, we further inferred that other features such as TFBS motifs could lead to the prediction of DeepHBV. HOMER was applied to recognize these TFBSs, and we found that these motifs might be related to HBV-related diseases or cancer development.

We noticed that the attention-intensive regions identified by the attention mechanism of DeepHBV with HBV integration sequences + TCGA Pan-Cancer strongly focused on the binding site of the *HIC1* tumor suppressor gene, the circadian clock-related elements BMAL1, CLOCK, c-Myc, NAPS2, and other transcription factors such as TEAD and Nr5a2. These DNA-binding proteins have been reported to be related to tumor development [27–33]. For instance, *HIC1* is a tumor suppressor gene, which is associated with hepatocarcinogenesis development [27, 28]. BMAL1, CLOCK, c-Myc, and NAPS2 are all related to the regulation of the circadian clock [29], which is closely related to HBV-related diseases [30, 31] (Additional file 4: Table S4). Together, these TFBSs on the human genome are closely associated with HBV integration, and their biological significance should be further verified by experimental research.

## Conclusion

This study developed an explainable attention-based deep learning model, DeepHBV, to predict HBV integration sites. DeepHBV is a robust deep learning model for predicting HBV integration sites and is the first attempt to use CNNs for HBV integration prediction. The attention mechanism in DeepHBV can be used to highlight the genomic preference for HBV integration and offer a deeper understanding of the mechanism underlying HBV-related cancer.

## Methods

### Data preparation

For DeepHBV model training and testing, 1000 bp DNA sequences were extracted from upstream and downstream, respectively, of HBV integration sites as a positive dataset. Each sample was denoted as $S=({n}_{1},{n}_{2},\dots ,{n}_{2000})$, where ${n}_{\mathrm{i}}$ represents the nucleotide in position *i*. DNA sequences do not contain HBV integration sites as negative samples. The existence of HBV integration hot spots, which contain several integration events within the 30–100,000 bp range [34], prompted us to select the background area with sufficient distance from known HBV integration sites. The regions within 50,000 bp around the known HBV integration sites in the hg38 reference genome were ignored. A 2000 bp sequence that did not contain HBV integration sites was randomly selected from the remaining regions as negative samples.

The extracted DNA sequences were encoded into one-hot code to calculate the similarity and distance between features in training more accurately. Original DNA sequences were converted to binary matrices of four dimensions, corresponding to one nucleotide type.

### Feature extraction

The DeepHBV model first applied convolution and pooling modules to learn and obtain sequence features around HBV integration sites (Additional file 1: Fig. S1). Specifically, the model employed multiple variant convolution kernels for the calculation to obtain different features. A DNA sequence is denoted as $S = \left({n}_{1},{n}_{2},\dots ,{n}_{2000}\right)$ and further encoded into a binary matrix *E*. Each binary matrix was entered into the convolution and pooling module for convolution calculations, according to $X=conv(E)$, which can be denoted as:

1 $$X_{{k,j}} = \mathop \sum \limits_{{j = 0}}^{{p - 1}} \mathop \sum \limits_{{l = 1}}^{L} W_{{k,j,l}} E_{{l,i + j}}$$

Here, 1 ≤ *k* ≤ *d*, *d* refers to the number of convolution kernels, 1 ≤ *j* ≤ *n* – *p* + 1, $j$ refers to the index, *p* refers to the convolution kernel size, n refers to the input sequence length, and $W$ refers to the convolution kernel weight.

The convolutional layer activated eigenvectors using a rectified linear unit (REL) after extracting relative eigenvectors and mapping each element on a sparse matrix. Next, the model applies a max-pooling strategy to minimize the dimensions and maximize the predicted information. The final eigenvector ${F}_{\mathrm{c}}$ was then extracted.

### The attention mechanism in the DeepHBV model

The attention mechanism was applied in DeepHBV to determine the contribution of each position to the extracted eigenvector ${F}_{\mathrm{c}}$. Each eigenvalue was assigned a weight value in the attention layer, which refers to the contribution level of the convolutional neural network (CNN) in that position.

The output from the convolution-and-pooling module, eigenvector ${F}_{\mathrm{c}}$, is the input of the attention layer, and the output is the weight vector $W$, which can be denoted as

2 $$W = att\left( {a_{1} ,a_{2} , \ldots ,a_{q} } \right)$$

Here, $att()$ refers to the attention mechanism, ${a}_{i}$ is the eigenvector in the ${i}^{th}$ dimension in the eigenmatrix, and $W$ refers to the dataset containing the contribution values of each position in the eigenmatrix extracted by the convolution-and-pooling module.

All contribution values were normalized to achieve a dense eigenvector matrix, which is denoted as ${F}_{a}$:

3 $$F_{a} = \mathop \sum \limits_{{j = 1}}^{q} a_{j} v_{j}$$

where ${a}_{j}$ refers to the relevant normalization value, and ${v}_{j}$ refers to the eigenvector at position $j$ of the input eigenmatrix. Each position refers to an extracted eigenvector in each convolution kernel.

The convolution-pooling module and the attention mechanism module must be combined in the model prediction. In other words, eigenvector ${F}_{c}$ and the relative eigenvalue ${F}_{a}$ should work together in predicting HBV integration sites.

The values in the eigenvector ${F}_{c}$ were linearly mapped to a new vector, ${F}_{v}$, which is

4 $$F_{v} = \left( {dense\left( {flatten\left( F \right)_{{\text{c}}} } \right)} \right)$$

In this step, the flattened layer performs the function $flatten()$ to reduce the dimension and concatenate data; the dense layer performed function $dense()$ to map dimension-reduced data to a single value. Then, the ${F}_{v}$ and ${F}_{a}$ concatenated vector entered the linear prediction classifier to calculate the probability that HBV integration occurred within the current sequence, as follows:

5 $$P = sigmoid\left( {concat\left( {F_{a} ,F_{v} } \right)} \right)$$

where $P$ is the predicted value, $sigmoid()$ refers to the activation function acting as a classifier in the final output, $concat()$ refers to the concatenation operation.

At the same time, the output eigenvector ${F}_{c}$ from the convolution-and-pooling module serves as the input and executes the attention mechanism where the weight vector $W$ can be described as:

6 $$W = att\left( {a_{1} ,a_{2} , \ldots ,a_{q} } \right)$$

Here, $W$ refers to the dataset containing the contribution values of each position in the eigenmatrix extracted by the convolution-and-pooling module, $att()$ refers to the attention mechanism, and ${a}_{i}$ refers to the eigenvector in the *i*th dimension in the eigenmatrix.

### DeepHBV model evaluation

After each parameter in DeepHBV was confirmed (Additional file 4: Table S1), the DeepHBV deep learning neural network model was trained using binary cross-entropy. The loss function of DeepHBV can be defined as:

7 $$Loss = - \mathop \sum \limits_{i} y_{i} \log \left( P \right) + \left( {1 - y_{i} } \right)\log \left( {1 - P} \right)$$

where ${y}_{i}$ is the prediction value, $P$ is the binary value of that sequence (in this dataset, positive samples were labeled as 1, and negative samples were labeled as 0).

To evaluate the best output of the DeepHBV model, a tenfold cross-validation was adopted. The confusion matrix, which included true positive, true negative, false positive, and false negative, followed by accuracy, specificity, sensitivity, AUROC, AUPR, Mathews’ correlation coefficient, and F-1 score, were adopted.

The DeepHBV model adapted Tensorflow1.13.1, scikit-learn0.24 [35] by NVIDIA Tesla V100-PCIE-32G (NVIDIA Corporation, California, USA).

## Supplementary Information

**Additional file 1.** Supplementary Figure 1.

**Additional file 2.** Supplementary Figure 2.

**Additional file 3.** Supplementary Notes.

**Additional file 4.** Supplementary Tables.

## Data Availability

DeepHBV is available as an open-source software and can be downloaded from: https://github.com/JiuxingLiang/DeepHBV.git

## References

[1] Liang TJ (2009). Hepatitis B: the virus and disease. Hepatology.

[2] Hai H, Tamori A, Kawada N (2014). Role of hepatitis B virus DNA integration in human hepatocarcinogenesis. World J Gastroenterol.

[3] Tu T, Budzinska MA, Shackel NA (2017). HBV DNA integration: molecular mechanisms and clinical implications. Viruses.

[4] Chami M, Gozuacik D, Saigo K (2000). Hepatitis B virus-related insertional mutagenesis implicates SERCA1 gene in the control of apoptosis. Oncogene.

[5] Koch S, von Loringhoven AF, Hofschneider PH (1984). Amplification and rearrangement in hepatoma cell DNA associated with integrated hepatitis B virus DNA. EMBO J.

[6] Steinemann D, Skawran B, Becker T (2006). Assessment of differentiation and progression of hepatic tumors using array-based comparative genomic hybridization. Clin Gastroenterol Hepatol.

[7] Sung WK, Zheng H, Li S (2012). Genome-wide survey of recurrent HBV integration in hepatocellular carcinoma. Nat Genet.

[8] Zhao LH, Liu X, Yan HX (2016). Genomic and oncogenic preference of HBV integration in hepatocellular carcinoma. Nat Commun.

[9] Ding D, Lou X, Hua D (2012). Recurrent targeted genes of hepatitis B virus in the liver cancer genomes identified by a next-generation sequencing-based approach. PLoS Genet.

[10] Tu T, Budzinska MA, Vondran FWR (2018). Hepatitis B virus dna integration occurs early in the viral life cycle in an in vitro infection model via sodium taurocholate cotransporting polypeptide-dependent uptake of enveloped virus particles. J Virol.

[11] Mason WS, Gill US, Litwin S (2016). HBV DNA integration and clonal hepatocyte expansion in chronic hepatitis B patients considered immune tolerant. Gastroenterology.

[12] LeCun Y, Bengio Y, Hinton G (2015). Deep learning. Nature.

[13] Litjens G, Kooi T, Bejnordi BE (2017). A survey on deep learning in medical image analysis. Med Image Anal.

[14] Bailey TL, Baker ME, Elkan CP (1997). An artificial intelligence approach to motif discovery in protein sequences: application to steroid dehydrogenases. J Steroid Biochem Mol Biol.

[15] Yamashita R, Nishio M, Do RKG (2018). Convolutional neural networks: an overview and application in radiology. Insights Imaging.

[16] Bahdanau D, Cho K, Bengio Y. Neural machine translation by jointly learning to align and translate. Computer Science 2014.

[17] Guidotti R, Monreale A, Ruggieri S (2018). A survey of methods for explaining black box models. ACM Comput Surv.

[18] Hu H, Xiao A, Zhang S (2019). DeepHINT: understanding HIV-1 integration via deep learning with attention. Bioinformatics.

[19] Haeussler M, Zweig AS, Tyner C (2019). The UCSC Genome Browser database: 2019 update. Nucleic Acids Res.

[20] Inoue F, Kircher M, Martin B (2017). A systematic comparison reveals substantial differences in chromosomal versus episomal encoding of enhancer activity. Genome Res.

[21] Robinson JT, Thorvaldsdottir H, Winckler W (2011). Integrative genomics viewer. Nat Biotechnol.

[22] Tang D, Li B, Xu T et al. VISDB: a manually curated database of viral integration sites in the human genome. Nucleic Acids Res 2019. 10.1093/nar/gkz867 PMC6943068 31598702

[23] Zhang W, Itoh K, Tanida J (1990). Parallel distributed processing model with local space-invariant interconnections and its optical architecture. Appl Opt.

[24] Bruna J, Zaremba W, Szlam A et al. Spectral networks and locally connected networks on graphs. Computer Science 2013.

[25] Heinz S, Benner C, Spann N (2010). Simple combinations of lineage-determining transcription factors prime cis-regulatory elements required for macrophage and B cell identities. Mol Cell.

[26] Seide F, Gang L, Dong Y. Conversational speech transcription using context-dependent deep neural networks. 2012.

[27] Taniguchi K, Roberts LR, Aderca IN (2002). Mutational spectrum of beta-catenin, AXIN1, and AXIN2 in hepatocellular carcinomas and hepatoblastomas. Oncogene.

[28] Zheng J, Xiong D, Sun X (2012). Signification of hypermethylated in cancer 1 (HIC1) as tumor suppressor gene in tumor progression. Cancer Microenviron.

[29] Paibomesai MI, Moghadam HK, Ferguson MM (2010). Clock genes and their genomic distributions in three species of salmonid fishes: associations with genes regulating sexual maturation and cell cycling. BMC Res Notes.

[30] Fekry B, Ribas-Latre A, Baumgartner C (2018). Incompatibility of the circadian protein BMAL1 and HNF4alpha in hepatocellular carcinoma. Nat Commun.

[31] Mukherji A, Bailey SM, Staels B (2019). The circadian clock and liver function in health and disease. J Hepatol.

[32] Huh HD, Kim DH, Jeong HS (2019). Regulation of TEAD transcription factors in cancer biology. Cells.

[33] Cai YN, Zhou Q, Kong YY (2003). LRH-1/hB1F and HNF1 synergistically up-regulate hepatitis B virus gene transcription and DNA replication. Cell Res.

[34] Hu Z, Zhu D, Wang W (2015). Genome-wide profiling of HPV integration in cervical cancer identifies clustered genomic hot spots and a potential microhomology-mediated integration mechanism. Nat Genet.

[35] Chollet Fao. Keras. 2015.

