# Supplementary figures and images for: DeepHBV: a deep learning model to predict hepatitis B virus (HBV) integration sites
Source: BMC Ecol Evol. 2021 Jul 7;21:138. doi: 10.1186/s12862-021-01869-8 (PMC8261932; doi:10.1186/s12862-021-01869-8)

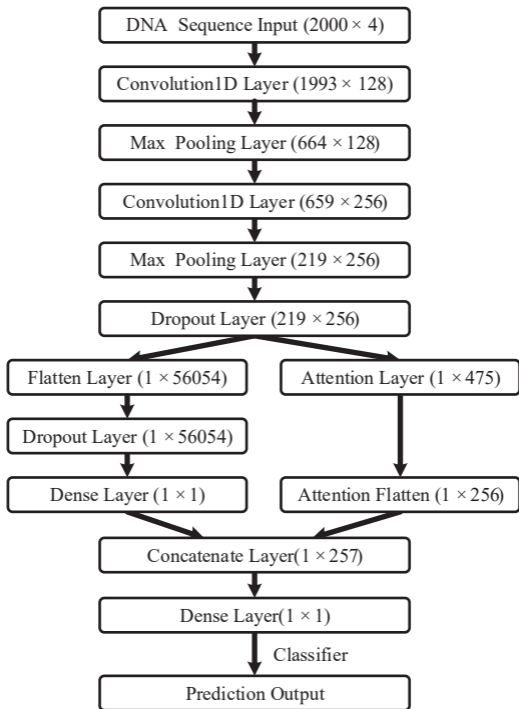

Supplement: Supplementary file 1 — Additional file 1. Supplementary Figure 1. [file 12862_2021_1869_MOESM1_ESM.pdf]

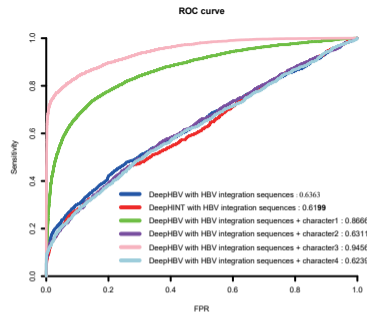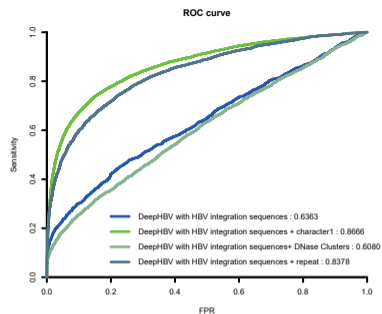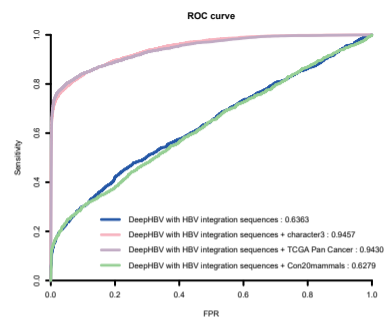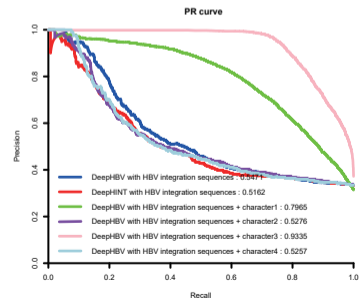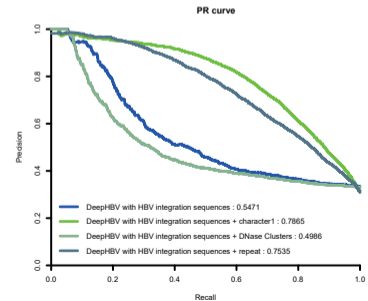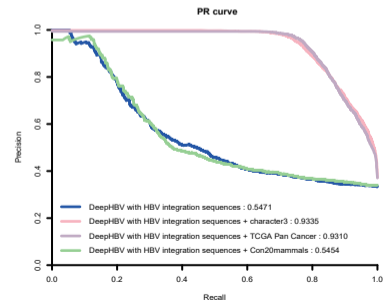

Supplement: Supplementary file 2 — Additional file 2. Supplementary Figure 2. [file 12862_2021_1869_MOESM2_ESM.pdf]
