# Supplementary material for: DeepHBV: a deep learning model to predict hepatitis B virus (HBV) integration sites
Source: BMC Ecol Evol. 2021 Jul 7;21:138. doi: 10.1186/s12862-021-01869-8 (PMC8261932; doi:10.1186/s12862-021-01869-8)
Supplement: Supplementary file 3 — Additional file 3. Supplementary Notes. [file 12862_2021_1869_MOESM3_ESM.docx]

**Supplementary Note**

## DeepHBV framework

### DeepHBV neural network structure design

This research established a series of deep learning neural network models with various structures, each model was evaluated using 30% of the positive samples and randomly selected negative samples. The model with best performance was selected to be the DeepHBV model (Supplementary Figure 1). This model is consisted with input layer, convolution1D layer, pooling layer, dropout layer, dense layer (fully connected layer) and attention layer, concatenate layer, classifier.

### 1.2 Hyperparameters involved in DeepHBV

The hyperparameters involved in DeepHBV are the kernel sized in Convolutional1D layer, kernel amount, the strategies to deal with sequences less than 2000 bp, activate function, constraint function applied on weights and bias-vectors, the combination length in max pooling layer, the hidden layer size and activation function in attention layer and the loss possibility in dropout layer.

## Mathematical matters of the DeepHBV

### 2.1 Encoding DNA sequences

DeepHBV adopted one-hot encoding function and represented each DNA sequence using matrix format. This step enables the model calculation more accurate on similarity and the distance between features in training. Each nucleotide was converted into a 4-bit length vector where each dimension represents one nucleotide type. As formula (2) showed, to a specific nucleotide type, the related dimension was set to 1 and the other dimensions were set to 0. If Ns existed in samples, we should set all dimensions into 0. After converting, we linked these binary vectors together as a 2000*4 matrix. If we describe this step in mathematical is: to a specific sequence S=($n_{1}$,$n_{2}$,…,$n_{2000}$), we can encoded the sequence using one-hot code as:

*E* = encoder(S) = (vector($n_{1}$),vector($n_{2}$),…,vector($n_{2000}$)) (1)

where，

vector($n_{i}$) = $\left\{ \begin{aligned} \left( 1,0,0,0 \right)，if n_{i}=A or n_{i}=a; \\ (0,1,0,0)，if n_{i}=T or n_{i}=t; \\ (0,0,1,0)，if n_{i}=C or n_{i}=c; \\ \left( 0,0,0,1 \right)，if n_{i}=G or n_{i}=g; \\ \left( 0,0,0,0 \right)，if n_{i}=N \end{aligned} \right.$ (2)

### 2.2 Convolution1D layers in DeepHBV

#### *2.2.1 Convolution kernels in convolution layers*

To input vector matrix, convolution layer can be calculated by *X*=$conv$($E$):

$X_{k,i}$= $\sum_{j=0}^{p-1} \sum_{l=1}^{L} W_{k,j,l}E_{l,i+j}$ (3)

Where, $1\leq k\leq d$, $d$ refers to the number of kernels (the first convolutional layer was set to 128, and the second convolutional layer was set to 256), $1\leq i\leq n-p+1$, $p$ refers to the kernel size (for 1^st^ and 2^nd^ convolution layer, kernel size is 8 and 6 respectively), $n$ refers to the input matrix length (2000 for 1^st^ convolution layer, 996 for 2^nd^ convolution layer), $W$ refers to the kernel weight,$L$ refers to the encoding vector dimension (4 in this model). $X_{k,i}$ stands for the score given by kernel $k$ when $k$ was aligned to $E$ at position i.

#### *2.2.2 Activation function in convolution layer*

We selected Rectified Linear Unit (ReLU) as the activation function in this research. Each element in the convolutional layer output was compared to the threshold 0, only those elements no less than the threshold was kept, any other element was set to 0 to erase the noise elements, which can be described by:

ReLU(x) = $\left\{ \begin{aligned} x, if x\geq0; \\ 0, if x<0. \end{aligned} \right.$ (4)

#### *2.2.3 Constraint function in convolution layer*

In order to limit over-fitting during training on DeepHBV, and make the model performs better, we added constraint function on network nodes during convolution layer optimisation. Constraint function constrained the main weight matrix in convolution layer using MaxNorm (Maximum norm weight constraint), which can be described as:

${||w||}_{2}$ < *m*  (5)

Where *m* is an adjustable parameter in MaxNorm, ${||.||}_{2}$ is L2 norm, $\left| \left| w \right| \right|$ have to be calculated in each training step in order to make sure it is less than *m*.

### 2.3 The Max Pooling Layer in DeepHBV

Max pooling layer keeps the most predicted information as well as reduces dimension, and decrease data amount to improve model calculating efficiency. DeepHBV applied the max pooling strategy. The calculation window size is 3. This progress can be described mathematically as: define the activation function output as Y, and the output of pooling as $F_{c}$=maxpooling(*Y*), which can be written by:

$F_{k,j}^{c}$ = max($Y_{k,i}$,$Y_{k,\left( i+1 \right)}$,$Y_{k,\left( i+2 \right)}$) (6)

Where$j$ is the index of the output position after pooling ($1\leq j\leq q$，$q$ refers to the summarize of sequence after pooling operation), i represents the index of the start positions; $k$ represents specific kernel size ($1 \leq k \leq d$, $d$ represents the width of pooling layer input vector, the output vector width was the same as input vector width). Therefore, the input vector can be translated into a $d\times q$ matrix as output (denoted as $F_{c}$).

### 2.4 Dropout layer in DeepHBV

DeepHBV added dropout layer, enabling a specific proportion of random neurons stop working to prevent overfitting (Srivastava et al. 2014), which can be presented by:

$v$ = *f*((*W*×$m$)×$F_{c}$) (7)

Where,

$m_{i,j}\sim Bernoulli(p)$ (8)

In which $f()$ is the activation function, $F_{c}$ is the input of dropout layer, *W* refers to the weight matrix. $v$ is the output from dropout layer, $m$ is the Dropout mask, the possibility of each element being 1 in Dropout mask is *p.*

### 2.5 Attention Layer in DeepHBV

After a series of operations eigenvector entered the attention layer, which gave these eigenvectors weight scores to identify the importance level. Attention layer transported each input vector into feedforward neural network contains single hidden layer (size was set to 256), the operation on eigenmatrix $F_{c}$ in this step can be described as:

$t$ = $W$ ($W_{0}$×$v$+$b_{0}$) + $b$ (9)

where $W_{0}$ (with size of $d\times n$ , $d$ is the number of convolution kernels (256)，$n$ is the size of hidden layer (256)),$W$ represents the weight matrix (with size 1×256), $b$, $b_{0}$ are related bias of DeepHBV deep learning neural network, $t$ is the importance score (dimension is $q$, where each element refers to the importance score in relevant position in $v$. Higher output weight score means that the site is more essential distribution of the relevant position to HBV integration sites prediction. The normalised importance scores as $a_{j}$ , the dense matrix $F_{a}$, then：

$F_{a}$ = $\sum_{j=1}^{q} a_{j}v_{j}$ (10)

$a_{j}$ = $\frac{exp(t_{j})}{\sum_{i}^{q} exp(t_{i})}$ (11)

where, $t_{j}$ stands for the importance score of that deep learning network output, $a_{j}$ represents the related normalised score, $v_{j}$ stands for eigenvalue of input eigenmatrix at position $j$.

### 2.6 Concatenate Layer in DeepHBV

The model prediction requires the convolution module output and the attention mechanism model output. DeepHBV concatenated the values of matrix $v$ and linearly mapped their values (denoted by $F_{v}$). Then concatenated $F_{v}$ with $F_{a}$:

*L* = $concat$($F_{a},F_{v}$) (12)

where $concat()$ denotes concatenate operation, define *A* = ($a_{1}$,$a_{2}$,…,$a_{n}$), *B* = ($b_{1}$,$b_{2}$,…,$b_{n}$), the progress can be described by:

$concat(A,B)$ = ($a_{1}$,$a_{2}$,…,$a_{n}$,$b_{1}$,$b_{2}$,…,$b_{n}$) (13)

### 2.7 Linear classifier in DeepHBV

To identify score $P$ of HBV integration probability, we used concatenated eigenmatrix $L$ as the input of Linear classifier, a sample will be identified to be positive when $P>0.5$:

$P$ = $sigmoid(L)$ =$\frac{1}{1+exp[-\left( W_{1}L+b_{W_{1}} \right)]}$ (14)

where $W_{1}$ represents the weight matrix of that deep learning neural network, $b_{W_{1}}$stands for the relevant bias, *L* stands for the eigenmatrix after concatenating.

### 2.8 Optimisation Algorithm

We trained DeepHBV deep learning neural network after tuning the hyperparameters using binary crossentropy, the loss function can be described as:

$loss$= -$\sum_{i} y_{i}\log\left( P \right)+\left( 1-y_{i} \right)\log\left( 1-P \right)$ (15)

where $y_{i}$ represents the prediction score, $P$ is the real binary tag of that sequence (the binary tag of positive samples is 1, and the binary tag of negative samples is 0 in this dataset. Back propagation algorithm was adapted in the training progress and Nesterov-accelerated adaptive moment estimation (Nadam) gradient descent algorithm to optimise parameter initialization. Nadam gradient descent algorithm is an improved version of Nesterov-accelerated gradient (NAG) algorithm in Adaptive moment estimation (Adam) [1], Nadam can be described as:

$g_{t}$=$\nabla_{\theta_{t-1}}f\left( \theta_{t-1} \right)$ (16)

$\hat{g}=\frac{g_{t}}{1-\Pi_{i=1}^{t}\mu_{i}}$ (17)

$m_{t}=\mu m_{t-1}+\left( 1-\mu\right)g_{t}$ (18)

$\hat{m}_{t}=\frac{m_{t}}{1-\Pi_{ⅈ=1}^{t+1}\mu_{i}}$ (19)

$n_{t}=\nu n_{t-1}+\left( 1-\nu\right)g_{t}^{2}$ (20)

$\hat{n}_{t}=\frac{n_{t}}{1-\nu^{t}}$ (21)

$\bar{m}_{t}=\left( 1-\mu_{t} \right)\hat{g}_{t}+\mu_{t}+1\hat{m}_{t}$ (22)

$\theta_{t}=\theta_{t-1}-\eta\frac{\bar{m}_{t}}{\sqrt{\hat{n}_{t}}+\varepsilon}$ (23)

## Pre-testing of region span and other parameters

DNA sequences with the length of 500 bp, 1,000 bp, 2,000 bp and 4,000 bp were pre-tested. When it was 2,000 bp, as is shown in Supplementary Table 5, the best performance was observed on most parameters such as accuracy, loss value, sensitivity, ROC and AUPR. In this case, the length of 2,000 bp was applied in the further study. Other parameters were set up in the same way, that is, by conducting thousands of iterative training and then comparing their index values such as loss value and accuracy. The eventually comprehensively selected optimal parameters are available in Table 1.

## Sigmoid classifier versus softmax classifier

Although both softmax and sigmoid can be used as two-class classifiers, we compared the performance of sigmoid and softmax as classifier. And the results showed that, sigmoid layer had a significant advantage of being the prediction classifier (Supplementary Table 6). So we used sigmoid as classifier in our model.

**Supplementary Table 6.** The performance of sigmoid and softmax as classifier

| classifier | loss | accuracy | sensitivity | specificity | AUROC | AUPR | F1-score | MCC |
| --- | --- | --- | --- | --- | --- | --- | --- | --- |
| softmax | 1.273 | 0.6952 | 0.6139 | 0.7057 | 0.6243 | 0.5837 | 0.3153 | 0.2161 |
| sigmoid | 1.1355 | 0.7368 | 0.7695 | 0.7321 | 0.6901 | 0.6012 | 0.4204 | 0.3521 |

AUROC, Area under receiver operating characteristic curve; AUPR, Area under precision-recall curve; MCC, Mathews’ correlation coefficient.

## Comparing DeepHBV with traditional machine learning models

We compared testing results of DeepHBV model with 3 traditional machine learning methods, Support Vector Machine (SVM), Logistic Regression (LR) and Random Forest (RF) in the internal and VISDB independent test dataset. Before training these machine learning models, we extracted 4 mononucleotides and 6 dinucleotide relative abundance (DRA) features (Karlin et al., Trends Genet, 1995). As is shown in the Supplementary Table 7, compared with traditional machine learning methods, DeepHBV exhibited better AUROC performance in both VISDB test dataset (DeepHBV = 0.7603, SVM = 0.5673, LR = 0.5149 and RF = 0.5836) and dsVIS test dataset (DeepHBV = 0.9430, SVM = 0.6371, LR = 0.6017 and RF = 0.6734).

**Supplementary Table 7.** The comparison of the testing results of DeepHBV with 3 traditional machine learning models in the VISDB and dsVIS test dataset

| test dataset | model | accuracy | sensitivity | specificity | AUROC | AUPR | F1-score | MCC |
| --- | --- | --- | --- | --- | --- | --- | --- | --- |
| VISDB test dataset | SVM | 0.6673 | 0.4917 | 0.6900 | 0.5673 | 0.5107 | 0.2525 | 0.1228 |
|  | LR | 0.6308 | 0.3704 | 0.6761 | 0.5149 | 0.4972 | 0.2293 | 0.0352 |
|  | RF | 0.6924 | 0.6040 | 0.7035 | 0.5836 | 0.5917 | 0.3044 | 0.2056 |
|  | DeepHBV | 0.7438 | 0.7804 | 0.7382 | 0.7603 | 0.6189 | 0.4476 | 0.3743 |
| dsVIS test dataset | SVM | 0.7187 | 0.6632 | 0.7287 | 0.6371 | 0.6274 | 0.4171 | 0.2988 |
|  | LR | 0.6902 | 0.5679 | 0.7125 | 0.6017 | 0.5733 | 0.3613 | 0.2152 |
|  | RF | 0.7517 | 0.7632 | 0.7496 | 0.6734 | 0.6781 | 0.4909 | 0.3963 |
|  | DeepHBV | 0.9111 | 0.9568 | 0.8946 | 0.9430 | 0.9310 | 0.8507 | 0.7985 |

AUROC, Area under receiver operating characteristic curve; AUPR, Area under precision-recall curve; MCC, Mathews’ correlation coefficient.

**Reference**

[Srivastava](https://xueshu.baidu.com/s?wd=author%3A%28N%20Srivastava%29%20&tn=SE_baiduxueshu_c1gjeupa&ie=utf-8&sc_f_para=sc_hilight%3Dperson) N., et al. Dropout: a simple way to prevent neural networks from overfitting. The Journal of Machine Learning Research 2014; 15(1): 1929–1958.

Karlin S., et al. Dinucleotide relative abundance extremes: a genomic signature. Trends Genet 1995; 11(7): 283-290. doi: 10.1016/s0168-9525(00)89076-9.
