# Supplementary material for: DeepHBV: a deep learning model to predict hepatitis B virus (HBV) integration sites
Source: BMC Ecol Evol. 2021 Jul 7;21:138. doi: 10.1186/s12862-021-01869-8 (PMC8261932; doi:10.1186/s12862-021-01869-8)
Supplement: Supplementary file 4 — Additional file 4. Supplementary Tables. [file 12862_2021_1869_MOESM4_ESM.docx]

**Supplementary Table 1.** The parameters for the deep neural network used in DeepHBV

| **Parameter** | **value** |
| --- | --- |
| Kernel number of the first convolution layer | 128 |
| Kernel size of the first convolution layer | 8 |
| Kernel number of the second convolution layer | 256 |
| Kernel size of the second convolution layer | 6 |
| The maximum norm constraint on weight in the convolution layer | 3 |
| Activation function in the convolution layer | ReLU |
| Padding 0 strategy in the convolution layer | Valid |
| The size of the pooling layer | 3 |
| Dropout rate between the pooling layer and attention layer | 0.55 |
| Dropout rate between flatten layer and dense layer | 0.5 |
| Hidden units in the attention layer | 256 |
| Activation function in the attention layer | Linear |
| Optimizer | Nadam |
|  |  |

**Supplementary Table 2.** Genomic features and sources (Access date: Novemember 16^th^, 2019)

| **Feature** | **Source** |
| --- | --- |
| DNase Clusters | UCSC Table Browser track: DNase Clusters |
| Fragile site | HumCFS: a database of fragile sites in human chromosomes <https://webs.iiitd.edu.in/raghava/humcfs/download.html> |
| RepeatMasker | UCSC Table Browser track: RepeatMasker |
| CpG islands | UCSC Table Browser track: CpG Islands |
| GeneHancer | UCSC Table Browser track: GeneHancer |
| Cons 20 Mammals | UCSC Table Browser track: Cons 20 Mammals |
| TCGA Pan-Cancer | UCSC Table Browser track: TCGA Pan-Cancer |
| H3K4Me3 ChIP-seq | ENCODE ENCFF728WLM |
| H3K27ac ChIP-seq | ENCODE ENCFF668WID |

**Supplementary Table 3.** Comparison of DeepHBV and DeepHINT result record

| **Source model** | **AUROC** | **AUPR** |
| --- | --- | --- |
| DeepHBV with HBV integration sequences | 0.6362542 | 0.547093 |
| DeepHBV with HBV integration sequences + character 1 | 0.8665945 | 0.796523 |
| DeepHBV with HBV integration sequences + character 2 | 0.6311194 | 0.527579 |
| DeepHBV with HBV integration sequences + character 3 | 0.9456537 | 0.93352 |
| DeepHBV with HBV integration sequences + character 4 | 0.6239043 | 0.525669 |
| DeepHBV with HBV integration sequences + repeat | 0.837821 | 0.753531 |
| DeepHBV with HBV integration sequences + TCGA Pan Cancer | 0.9429895 | 0.930981 |
| DeepHINT with HBV integration sequences | 0.6199308 | 0.516248 |
| DeepHINT with HBV integration sequences + character 1 | 0.5777409 | 0.408657 |
| DeepHINT with HBV integration sequences + character 2 | 0.6124292 | 0.499724 |
| DeepHINT with HBV integration sequences + character 3 | 0.768824 | 0.707779 |
| DeepHINT with HBV integration sequences + character 4 | 0.6083628 | 0.49137 |
| DeepHBV with HBV integration sequences + repeat +  (independent test) VISDB | 0.6656823 | 0.573663 |
| DeepHBV with HBV integration sequences + TCGA Pan Cancer + (independent test) VISDB | 0.7603363 | 0.618921 |

**Supplementary Table 4.** Enriched TFBS from attention intensive regions of DeepHBV with HBV integration sites + repeat peaks

| **HOMER known results** | | |  | **HOMER de novo results** | | |
| --- | --- | --- | --- | --- | --- | --- |
| **Rank** | **Name** | **P-value** |  | **Rank** | **Best Match/Details** | **P-value** |
| 1 | Pitx1 | 1E-502 |  | 1 | Nr2e3 | 1E-4210 |
| 2 | CRX | 1.00E-208 |  | 2 | AP-1 | 1E-2791 |
| 3 | AP-1 | 1.00E-131 |  | 3 | Tbp | 1E-2189 |
| 4 | GSC | 1.00E-88 |  | 4 | EBF3 | 1E-1924 |
| 5 | BATF | 1.00E-76 |  | 5 | RHOXF1 | 1E-1433 |
| 6 | NPAS | 1.00E-65 |  | 6 | Zfp128 | 1E-890 |
| 7 | BMAL1 | 1.00E-56 |  | 7 | RHOXF1 | 1E-525 |
| 8 | JunB | 1.00E-50 |  | 8 | TFE3 | 1E-519 |
| 9 | Foxo3 | 1.00E-49 |  | 9 | TEAD3 | 1E-518 |
| 10 | Bach2 | 1.00E-36 |  | 10 | GLIS3 | 1E-435 |
| 11 | RARa | 1.00E-31 |  | 11 | RBPJ | 1E-361 |
| 12 | ZNF416 | 1.00E-25 |  | 12 | MEIS1 | 1.00E-285 |
| 13 | Otx2 | 1.00E-21 |  | 13 | YY2 | 1.00E-224 |
| 14 | Bapx1 | 1.00E-21 |  | 14 | Pknox2 | 1.00E-163 |
| 15 | Pit1 | 1.00E-18 |  | 15 | ZBTB6 | 1.00E-150 |
| 16 | HIF-1b | 1.00E-13 |  | 16 | PSE | 1.00E-93 |
| 17 | MNT | 1.00E-13 |  | 17 | STAT6 | 1.00E-87 |
| 18 | CEBP | 1.00E-13 |  |  |  |  |
| 19 | Foxo1 | 1.00E-11 |  |  |  |  |
| 20 | Oct11 | 1.00E-10 |  |  |  |  |
| 21 | TEAD | 1.00E-09 |  |  |  |  |
| 22 | ZNF519 | 1.00E-08 |  |  |  |  |
| 23 | Rbpj1 | 1.00E-08 |  |  |  |  |
| 24 | CHR | 1.00E-07 |  |  |  |  |
| 25 | IRF4 | 1.00E-07 |  |  |  |  |
| 26 | Oct4 | 1.00E-06 |  |  |  |  |
| 27 | Stat3 | 1.00E-06 |  |  |  |  |
| 28 | TEAD4 | 1.00E-06 |  |  |  |  |
| 29 | c-Myc (LNCAP-cMyc-ChIP-Seq) | 1.00E-06 |  |  |  |  |
| 30 | FOXA1 (MCF7-FOXA1-ChIP-Seq) | 1.00E-05 |  |  |  |  |
| 31 | FOXA1(LNCAP-FOXA1-ChIP-Seq) | 1.00E-05 |  |  |  |  |
| 32 | MafA | 1.00E-04 |  |  |  |  |
| 33 | CLOCK | 1.00E-04 |  |  |  |  |
| 34 | Mef2a | 1.00E-04 |  |  |  |  |
| 35 | TEAD1 | 1.00E-04 |  |  |  |  |
| 36 | MITF | 1.00E-04 |  |  |  |  |
| 37 | Tbr1 | 1.00E-04 |  |  |  |  |
| 38 | NPAS2 | 1.00E-03 |  |  |  |  |
| 39 | Oct2 | 1.00E-03 |  |  |  |  |
| 40 | STAT6 | 1.00E-03 |  |  |  |  |
| 41 | NFAT | 1.00E-03 |  |  |  |  |
| 42 | FOXK1 | 1.00E-03 |  |  |  |  |
| 43 | Erra | 1.00E-03 |  |  |  |  |
| 44 | TEAD2 | 1.00E-03 |  |  |  |  |
| 45 | HEB | 1.00E-03 |  |  |  |  |
| 46 | Gfi1b | 1.00E-02 |  |  |  |  |
| 47 | Arnt:Ahr | 1.00E-02 |  |  |  |  |
| 48 | bHLHE40 | 1.00E-02 |  |  |  |  |
| 49 | Olig2 | 1.00E-02 |  |  |  |  |
| 50 | c-Myc(mES-cMyc-ChIP-Seq) | 1.00E-02 |  |  |  |  |

**Supplementary Table 5.** The performance of DNA sequence samples of different lengths

| length/bp | loss | accuracy | sensitivity | specificity | AUROC | AUPR | F1-score | MCC |
| --- | --- | --- | --- | --- | --- | --- | --- | --- |
| 500 | 0.9759 | 0.6989 | 0.6494 | 0.7047 | 0.6015 | 0.4870 | 0.3018 | 0.2257 |
| 1000 | 1.1634 | 0.7195 | 0.7117 | 0.7207 | 0.6499 | 0.5476 | 0.3747 | 0.2967 |
| 2000 | 1.1355 | 0.7368 | 0.7695 | 0.7321 | 0.6901 | 0.6012 | 0.4204 | 0.3521 |
| 4000 | 1.2065 | 0.7213 | 0.6994 | 0.7246 | 0.6516 | 0.5534 | 0.3959 | 0.3036 |

AUROC, Area under receiver operating characteristic curve; AUPR, Area under precision-recall curve; MCC, Mathews’ correlation coefficient.

**Supplementary Table 6.** The performance of sigmoid and softmax as classifier

| classifier | loss | accuracy | sensitivity | specificity | AUROC | AUPR | F1-score | MCC |
| --- | --- | --- | --- | --- | --- | --- | --- | --- |
| softmax | 1.273 | 0.6952 | 0.6139 | 0.7057 | 0.6243 | 0.5837 | 0.3153 | 0.2161 |
| sigmoid | 1.1355 | 0.7368 | 0.7695 | 0.7321 | 0.6901 | 0.6012 | 0.4204 | 0.3521 |

AUROC, Area under receiver operating characteristic curve; AUPR, Area under precision-recall curve; MCC, Mathews’ correlation coefficient.

**Supplementary Table 7.** The comparison of the testing results of DeepHBV with 3 traditional machine learning models in the VISDB and dsVIS test dataset

| test dataset | model | accuracy | sensitivity | specificity | AUROC | AUPR | F1-score | MCC |
| --- | --- | --- | --- | --- | --- | --- | --- | --- |
| VISDB test dataset | SVM | 0.6673 | 0.4917 | 0.6900 | 0.5673 | 0.5107 | 0.2525 | 0.1228 |
|  | LR | 0.6308 | 0.3704 | 0.6761 | 0.5149 | 0.4972 | 0.2293 | 0.0352 |
|  | RF | 0.6924 | 0.6040 | 0.7035 | 0.5836 | 0.5917 | 0.3044 | 0.2056 |
|  | DeepHBV | 0.7438 | 0.7804 | 0.7382 | 0.7603 | 0.6189 | 0.4476 | 0.3743 |
| dsVIS test dataset | SVM | 0.7187 | 0.6632 | 0.7287 | 0.6371 | 0.6274 | 0.4171 | 0.2988 |
|  | LR | 0.6902 | 0.5679 | 0.7125 | 0.6017 | 0.5733 | 0.3613 | 0.2152 |
|  | RF | 0.7517 | 0.7632 | 0.7496 | 0.6734 | 0.6781 | 0.4909 | 0.3963 |
|  | DeepHBV | 0.9111 | 0.9568 | 0.8946 | 0.9430 | 0.9310 | 0.8507 | 0.7985 |

AUROC, Area under receiver operating characteristic curve; AUPR, Area under precision-recall curve; MCC, Mathews’ correlation coefficient.

**Supplementary Table 8.** The testing confusion matrix of DNA sequence samples of 2000 bp length

| Flod No. | predict lable  true lable | 1 | 0 |
| --- | --- | --- | --- |
| Flod No.1 | 1 | 354 | 93 |
|  | 0 | 689 | 2015 |
| Flod No.2 | 1 | 337 | 90 |
|  | 0 | 706 | 2017 |
| Flod No.3 | 1 | 333 | 94 |
|  | 0 | 709 | 2014 |
| Flod No.4 | 1 | 224 | 81 |
|  | 0 | 818 | 2015 |
| Flod No.5 | 1 | 245 | 92 |
|  | 0 | 797 | 2016 |
| Flod No.6 | 1 | 257 | 72 |
|  | 0 | 785 | 2036 |
| Flod No.7 | 1 | 343 | 112 |
|  | 0 | 699 | 1996 |
| Flod No.8 | 1 | 325 | 75 |
|  | 0 | 717 | 2033 |
| Flod No.9 | 1 | 275 | 102 |
|  | 0 | 767 | 2006 |
| Flod No.10 | 1 | 335 | 86 |
|  | 0 | 707 | 2022 |
